# The responsively decreased PKM2 facilitates the survival of pancreatic cancer cells in hypoglucose

**DOI:** 10.1038/s41419-017-0158-5

**Published:** 2018-01-26

**Authors:** Xiang Li, Shichang Deng, Mingliang Liu, Yan Jin, Shuai Zhu, Shijiang Deng, Jingyuan Chen, Chi He, Qi Qin, Chunyou Wang, Gang Zhao

**Affiliations:** 1 Department of Pancreatic Surgery, Union Hospital, Tongji Medical College, Huazhong University of Science and Technology, Wuhan, 430022 China; 2 Department of Gastrointestinal Surgery, Union Hospital West Campus, Tongji Medical College, Huazhong University of Science and Technology, Wuhan, 430056 China

## Abstract

Cancer cells predominantly produce energy at a high rate of glycolysis even in aerobic environment. It is termed as Warburg effect and is necessary for the tumorigenesis. Studies showed pyruvate kinase M2 (PKM2), a key regulator of the Warburg effect, is overexpressed and involved in numerous cancers. However, the expression and function of PKM2 in pancreatic cancer (PC) remain undefined. Our results showed that PKM2 is overexpressed in the PC tissue compared to the peritumoral tissue. Unexpected, the downregulation of PKM2 did not affect the proliferation, invasion, and chemoresistance of PC cells. Since pancreatic cancer is a hypovascular tumor with comparably insufficient energy supply, we further investigate the relationship between PKM2 and hypoglucose. Interestingly, we further discovered that decreased expression of PKM2 was detected in PC samples with lower microvessel density as well as in PC cells treated with hypoglucose condition (0.5 mM). Furthermore, the downregulation of PKM2 facilitated, while the upregulation of PKM2 inhibited, PC cells survival during hypoglucose. We further revealed that the repressed PKM2 induced autophagy, high NADPH/NADP ratio, and biomacromolecule production, but reduced ROS accumulation. Moreover, AMPKα1 knockdown repressed the autophagy and survival of PC cells during hypoglucose, which were promoted by PKM2 knockdown. Collectively, our study indicates that decreased PKM2 diverts glucose metabolism to biomacromolecule accumulation and antioxidants generation during glucose deprivation. This metabolism alteration elevates AMPKα1-dependent autophagy, which facilitates PC cell survival during glucose deprivation. Therefore, functions of PKM2 are complicated and cannot be defined as oversimplified promoter or inhibitor in PC.

## Introduction

Research demonstrates that cancer cells employ energy at a high rate of anaerobic glycolysis even in aerobic condition, which is called Warburg effect^1–3^. Pyruvate kinase M2 (PKM2), which functions like a gatekeeper of glycolysis and metabolic flux distribution^4^, is considered as a key regulator of the Warburg effect^5,6^. PKM2 regulates cell proliferation by modulating the intercellular concentration of ATP and PEP as a tap of glucose metabolism. PKM2 is characteristic of cells with high rates of nucleic acid synthesis, especially in cancer cells^7,8^. In rapidly proliferating cancer cells, PKM2 intermediates the synthesis of cell components by inducing the transformation of phosphoenolpyruvate (PEP) to pyruvate, which is totally independent of oxygen^9^.

PKM2 is involved in not only metabolic regulation but also tumorigenesis of tumor cells. PKM2 participates in regulation of gene transcription by interacting with HIF-1, Oct-4, STAT3, and β-catenin^4,10–14^. Research reveals that downregulation of PKM2 could inhibit proliferation and promote apoptosis in breast cancer, liver cancer, and gastric cancer cells^15–17^. Christofk et al.^18^ demonstrate that downregulating PKM2 with RNAi and replacing it with PKM1 led to a reversal of the Warburg effect and reduced the ability to form tumors in nude mouse xenografts. Apparently, these results indicate that PKM2 might act as a promoter in progression of numerous tumors. While differently, the other study demonstrates contradictory results. Cortéscros et al.^19^ reveal that the size of nude xenograft tumors was unaffected after PKM2 knockdown, which indicated that PKM2 was dispensable for tumor growth and maintenance especially in vivo. Moreover, the other research provides evidence that binding of phosphotyrosine peptides to PKM2 led to inhibition of PKM2 enzymatic activity, which supports rapid growth in cancer cells^18^. Meanwhile, the finding from Anastasiou et al.^20^ indicates that binding of activators to PKM2 promoted a constitutively active enzyme state which inhibited the growth of H1299 lung cancer cells both in vitro and in vivo. Therefore, these contradict results imply that the function of PKM2 in cancer is multifaceted and complex, as well as heterogenetic in different cancers.

Recently, Joergensen et al.^21^ demonstrate that the PKM2 level in plasma of pancreatic cancer (PC) patients was obviously highly expressed and strongly correlated with poor outcome. It suggests that plasma PKM2 could be a novel biological marker of PC. Moreover, research of Feng et al.^22^ illustrate that the PKM2 was overexpressed in local PC tumor mass and acted as a promoter in tumorigenesis. However, Aloysius et al.^23^ found that PKM2 was present only in benign non-ductal epithelium in normal pancreas and peritumoral tissue, but not in benign pancreatic ducts, premalignant lesions, and cancer. These paradoxical results suggest that the expression of PKM2 in PC tissues remains controversial and its underlying effects require improved understanding. Hence, it is worth to clarify the differential expression of PKM2 between PC and paired adjacent normal pancreatic (NP) tissues and further investigate its function.

PKM2 activity is a result of tumor environment and responsible for maintaining a glycolytic phenotype for cancer cell metabolism. The less active form of PKM2 leads to accumulation of glycolytic intermediates, which are available as precursors for biosynthetic processes such as amino acid, nucleic acid, and phospholipid anabolism^24^. Such glucose flux shifting from energy production to phosphogluconate pathway (PPP) can facilitate the proliferation of cells^7^. Since PC is a relatively hypovascular solid tumor which is characterized by intensive interstitial fibrosis^25^, thus the environment in which PC cells live comparatively remains energy insufficient. Taking this energy-insufficient environment and the pivotal role of PKM2 in glycolytic into consideration, it drew our remarkable interest to uncover the role of PKM2 in PC cells under hypoglucose condition.

## Results

### PKM2 is remarkably overexpressed in PC tissues

**Fig. 1:**
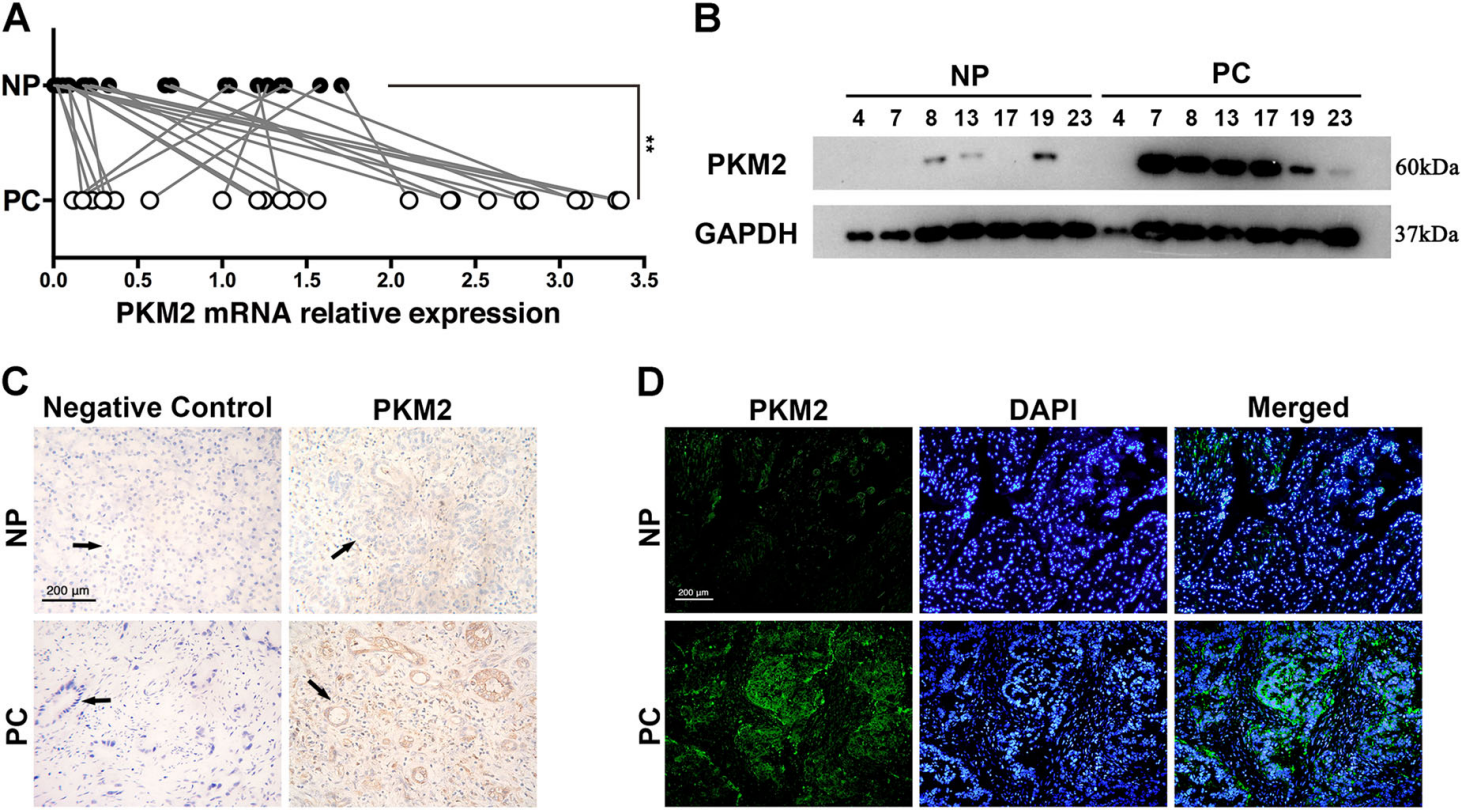
Analysis of PKM2 expression in human pancreatic cancer tissues **a** QRT-PCR was used to identify the expression of PKM2 mRNA in human pancreatic cancer tissues (PC) and adjacent normal pancreatic tissues (NP). **b** Western blot analysis displayed the differential expression of PKM2 in 7 out of 24 paired PC and peritumoral tissues. **c** Immunohistochemical analysis of PKM2 expression in representative PC and peritumoral sample is shown. The duct is directed by a black arrow. **d** Immunofluorescence analysis of PKM2 expression in representative PC and peritumoral sample **p < 0.01

Twenty-four cases of PC and paired adjacent NP tissues were used to identify the aberrant expression of PKM2. The data of quantitative real-time reverse transcription polymerase chain reaction (qRT-PCR) demonstrated that PKM2 was substantially increased in PC tissues at mRNA level (Fig. 1a). Western blot was carried out in 7 out of 24 paired PC and peritumoral samples to compare the expression level of PKM2 (Fig. 1b). Moreover, immunohistochemical stain and immunofluorescence were also applied for the expression level of PKM2 (Fig. 1c and d).

### Knockdown of PKM2 has no significant effects on proliferation, chemosensitivity, and invasion of PC cells

SiRNA-mediated knockdown of PKM2 caused an obvious drop of PKM2 expression in both PANC-1 (39.2%) and BXPC-3 (47.6%) cells, and led to inhibition of PKM2 activity as well (SFig. 1A and B). Unexpectedly, the PKM2 downregulation failed to effect proliferation in PC cells (SFig. 1C). Moreover, the invasion ability of PC cells was unaffected after transfection with siPKM2 either (SFig. 1D). On the other hand, the IC_50_ of both PANC-1 and BXPC-3 cells treated with siPKM2 was not significantly changed during treatment with 5-Fluorouracil (5-FU) and Gemcitabine (SFig. 1E).

### PKM2 expression is decreased in hypovascular area of PC tissues and PC cells treated with hypoglucose

**Fig. 2:**
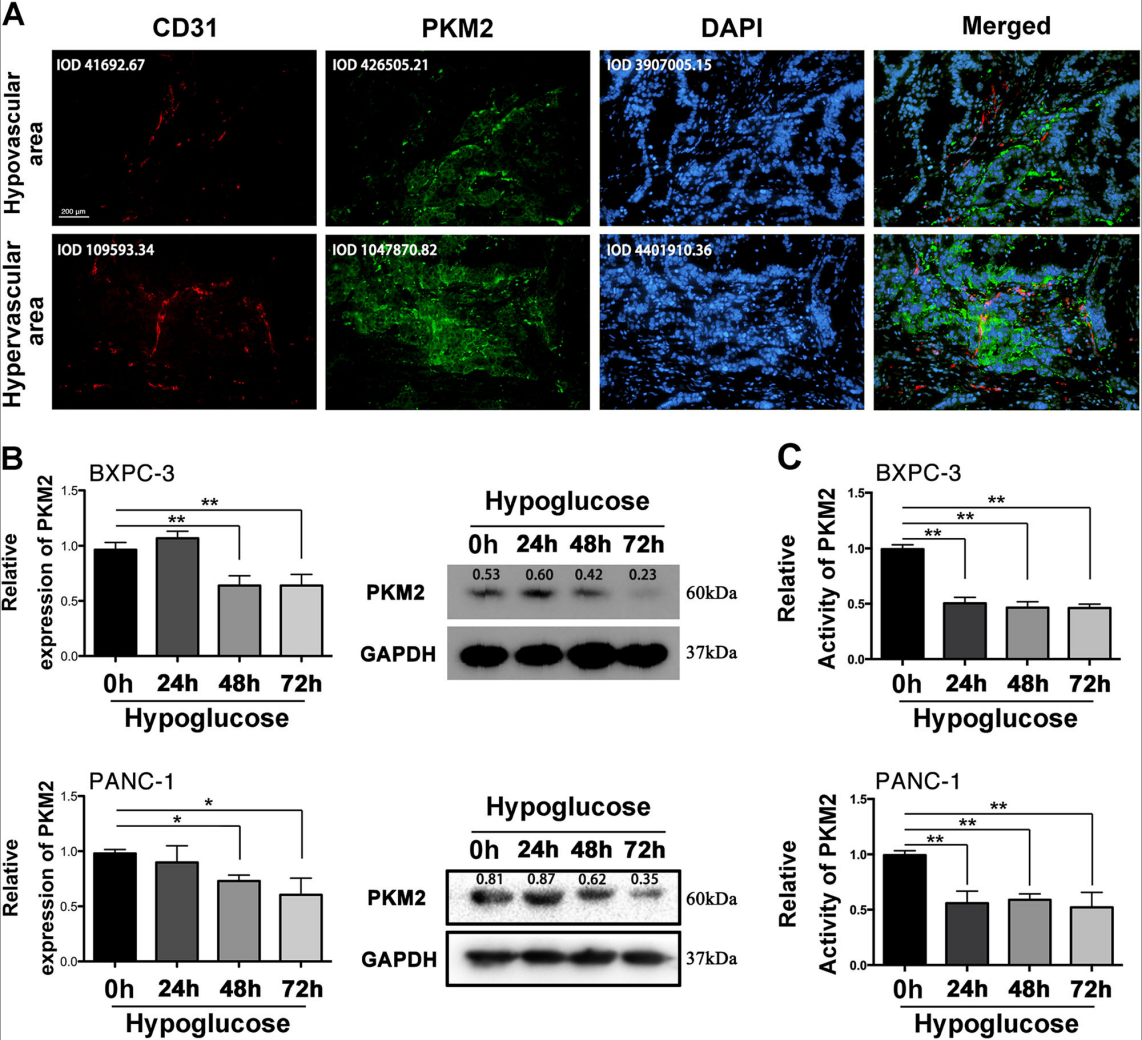
Downregulated PKM2 was observed in hypovascular area of PC and hypoglucose condition **a** Immunofluorescence analysis of PKM2 and CD31 expression in representative pancreatic tissue sample is shown. Integrated optic density (IOD) values are marked. **b** QRT-PCR and western blot were performed to detect the expression of PKM2 treated with hypoglucose (0.5 mM). **c** The relative PKM2 activity was detected after being treated with hypoglucose (*p < 0.05; **p < 0.01)

The microenvironment in which PC cells live remains energy insufficient, hence we further explored whether there was a link between energy status and PKM2 expression. As shown in Fig. 2a, three human PC tissues were examined under a microscope in five fields. All pictures were divided into two groups according to the mean integrated optic density (IOD) value of CD31. The average IOD values of CD31 in hypervascular and hypovascular groups are 41692.67 and 109593.34, respectively. We did find that the intensity of green fluorescence (PKM2) (average IOD values in two groups are 426505.21 and 1047870.82) was positively correlated with that of red one (CD31, to measure the density of vessel) in PC tumor mass, which implied that relative lower expressed PKM2 might be caused by insufficient energy. Furthermore, we identified the reduction of PKM2 expression in BXPC-3 and PANC-1 treated with hypoglucose by performing qRT-PCR and western blot (Fig. 2b). Moreover, the PKM2 activity in PC cells was also reduced after treatment with hypoglucose medium (Fig. 2c).

### Decreased PKM2 facilitates survival of PC cells in hypoglucose microenvironment

**Fig. 3:**
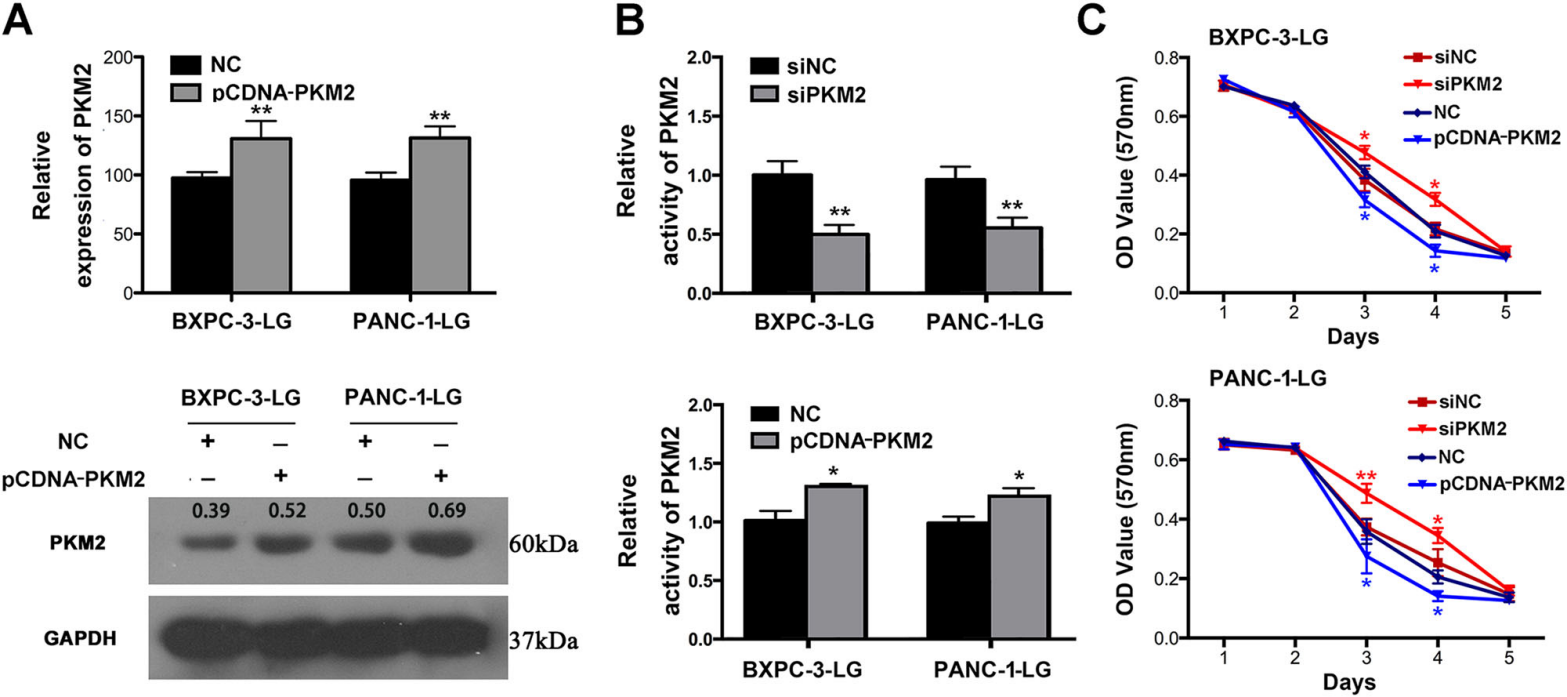
The effects of PKM2 on cell viability of pancreatic cancer cells in the hypoglucose medium **a** QRT-PCR and western blot were performed to determine PKM2 expression after transfection with PKM2-overexpressed plasmid (pCDNA-PKM2) and NC in BXPC-3-LG and PANC-1-LG (cells cultured with hypoglucose condition). **b** The activity of PKM2 was tested in both BXPC-3-LG and PANC-1-LG. **c** MTT assay was performed to measure the effects of PKM2 on proliferation (*P < 0.05; **P < 0.01)

We further identified the role of PKM2 on the survival of PC cells in hypoglucose microenvironment. Transfection with pcDNA-PKM2 leads to significantly increased mRNA level of PKM2 in BXPC-3-LG (32.3%) and PANC-1-LG (33.4%) cells (LG, cultured with hypoglucose medium), as well as protein expression (Fig. 3a). PKM2 knockdown further reduced PKM2 activity, while PKM2 upregulation elevated its activity in hypoglucose treatment (Fig. 3b). After downregulation of PKM2, both the survival of BXPC-3-LG and PANC-1-LG cells were distinctly promoted, while pcDNA-PKM2 leads to survival inhibition (Fig. 3c). However, little effects of PKM2 on chemoresistance and cell invasion of PC under hypoglucose treatment were observed (SFig. 2).

### PKM2 could regulate the expression of p-AMPKα1, AMPKα1, p-AKT, and SIRT1, lactic acid production, and ROS accumulation of PC cells in hypoglucose microenvironment

Since PKM2 is the key regulator of glucose metabolism, we further identified whether decreased PKM2 could control the glucose flux to induce precursors for biosynthetic processes instead of energy production. The expression of p-AMPKα1, AMPKα1, p-AKT, and SIRT1 of BXPC-3 cells was upregulated in hypoglucose treatment which was further increased after downregulation of PKM2, but decreased by PKM2 overexpression (SFigs. 3A and 4A). With hypoglucose treatment, decreased concentration of lactic acid and increased oxidative stress level were observed in BXPC-3 cancer cells. Moreover, both lactic acid production and oxidative stress level was increased after PKM2 overexpression (SFig. 3B and D), but downregulated after transfection with siPKM2 under low glucose condition (SFig. 4B and D). Moreover, the ratio of NADPH/NADP was decreased during hypoglucose, which was further decreased by PKM2 overexpression (SFig. 3C). On the contrary, PKM2 knockdown increased the NADPH/NADP ratio during hypoglucose (SFig. 4C). The similar results were observed in PANC-1 cell line (SFigs. 5 and 6). These results further indicated that the decreased PKM2 expression in hypoglucose changed the glucose flux to PPP, leading to biomolecule accumulation and antioxidants generation.

### Hypoglucose induces remarkable autophagy in PC cells

**Fig. 4:**
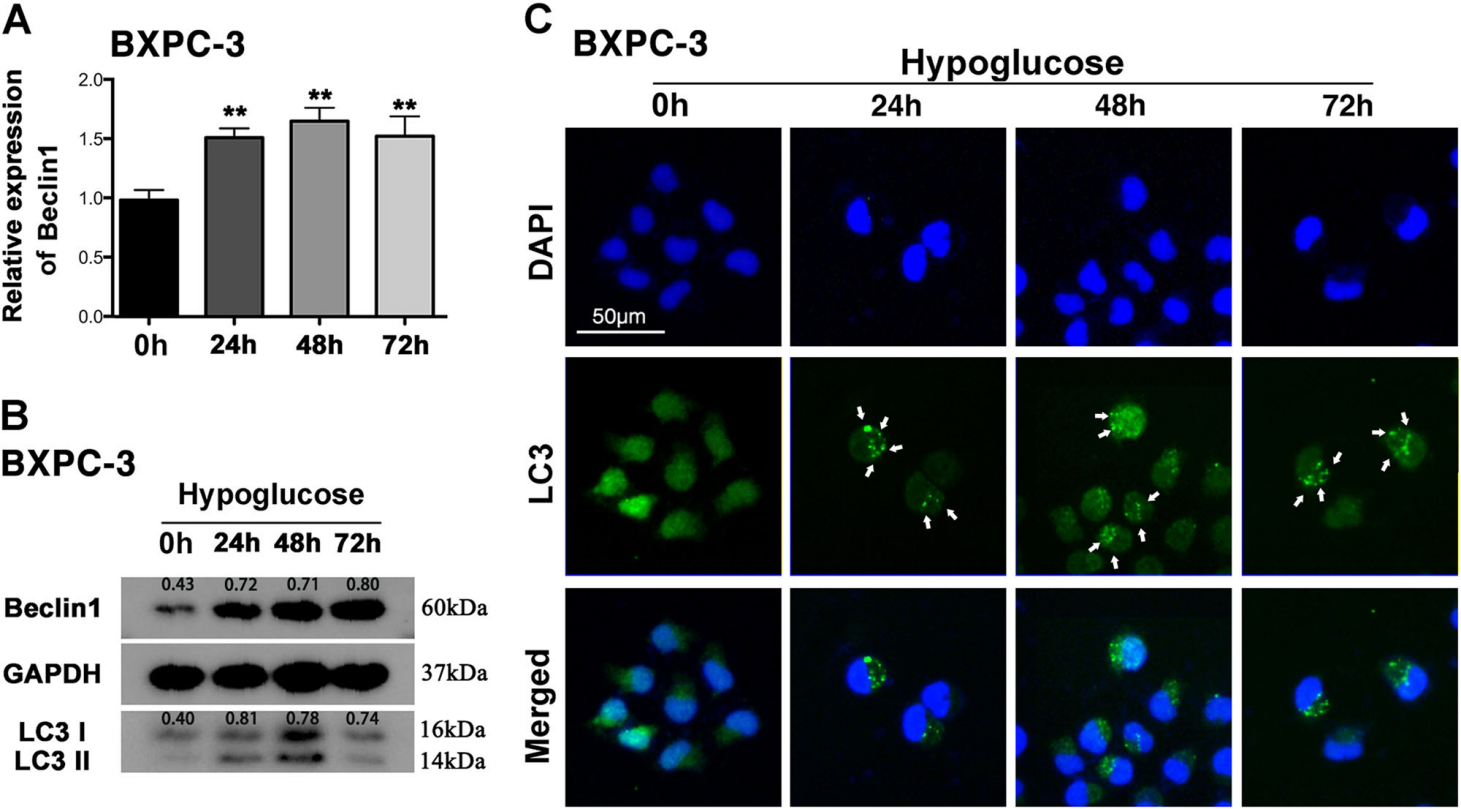
Hypoglucose treatment induced autophagy in BXPC-3 **a** QRT-PCR tested the mRNA of Beclin1 in BXPC-3 treated with hypoglucose medium for 0, 24, 48, and 72 h. **b** The result of western blot showed the expression of AMPKα1, Beclin1, and LC3II/LC3I. **c** Treatment of BXPC-3 with hypoglucose showed increased autophagosomes in cytoplasm as indicated by the arrow **P < 0.01

After treated with hypoglucose medium, the obvious increase of AMPKα1, Beclin1, and autophagy marker microtubule-associated protein light chain 3 (LC3) both in mRNA and protein level was observed (Fig. 4a and b). The density ratio of LC3II/LC3I also was increased in 24, 48, and 72 h compared to 0 h group (Fig. 4b). In order to further determine the autophagy intensity, we performed the immunofluorescence assay to identify the expression of LC3. As shown in Fig. 4c, more autophagosomes in the cytoplasm of BXPC-3 were detected under a fluorescence microscope after cells cultured with low glucose medium, indicating obviously increased autophagy level. The similar results were observed in PANC-1 cultured with hypoglucose (SFig. 7).

### Decreased PKM2 contributes to hypoglucose-induced autophagy in PC cells by upregulating AMPKα1

**Fig. 5:**
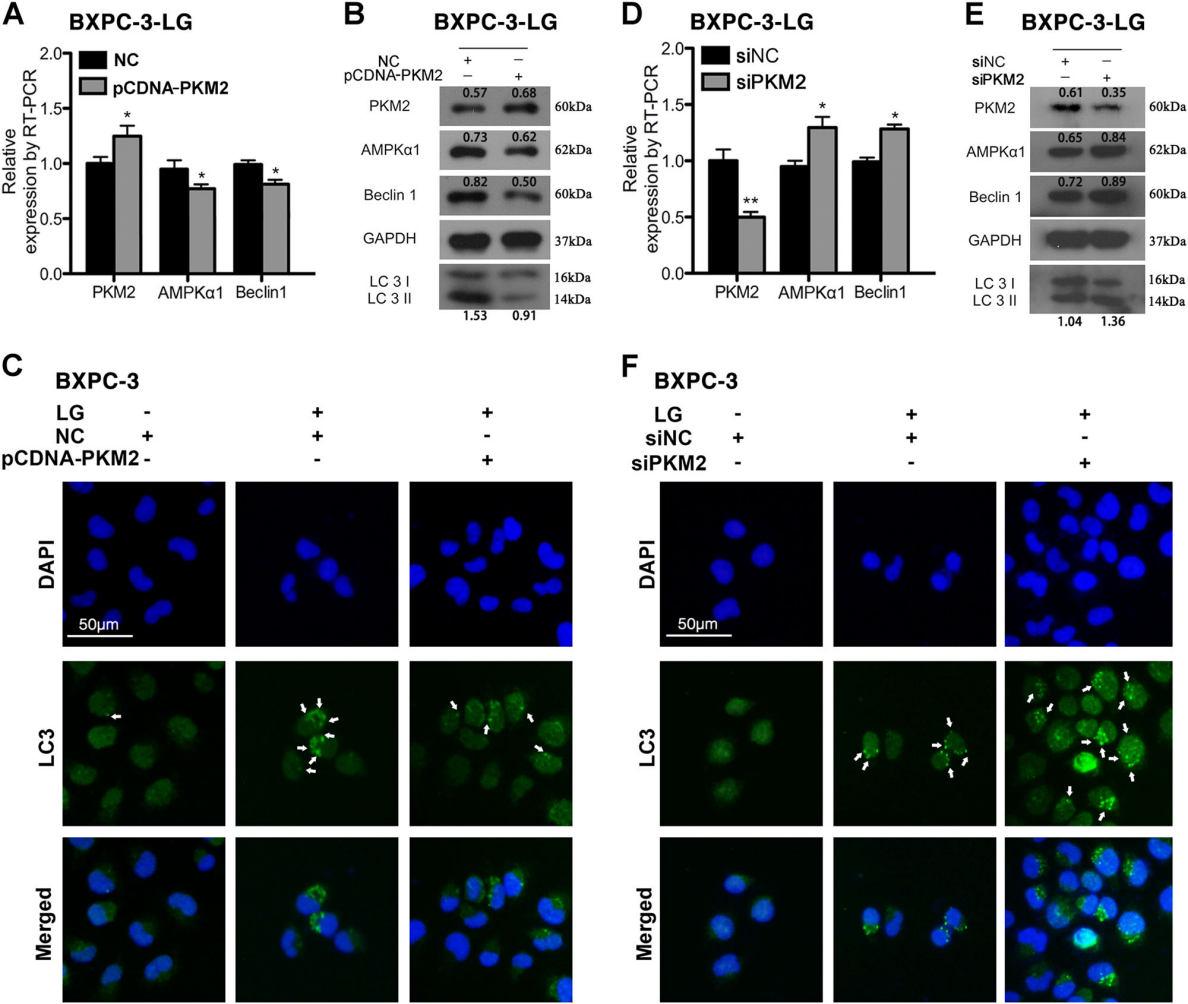
Decreased PKM2 promoted autophagy of BXPC-3 cells in hypoglucose by upregulating AMPKα1 expression **a** The expression level of PKM2, AMPKα1, and Beclin1 was tested by qRT-PCR after transfection with pCDNA-PKM2 in BXPC-3-LG. **b** The expression of PKM2, AMPKα1, and autophagy-related protein was determined by western blot. **c** Immunofluorescence revealed the autophagic level of pCDNA-PKM2-transfected cells in hypoglucose condition. **d** The expression level of PKM2, AMPKα1, and Beclin1 was tested by qRT-PCR after transfection with siPKM2 in BXPC-3-LG. **e** The expression of PKM2, AMPKα1, and autophagy-related protein was determined by western blot after transfection with siPKM2 in BXPC-3-LG. **f** Immunofluorescence revealed the autophagic level of siPKM2-transfected cells in hypoglucose condition. The autophagosome was indicated with the arrow (*P < 0.05; **P < 0.01)

Both qRT-PCR and western blot results demonstrated that overexpressed PKM2 significantly decreased Beclin1 expression, density ratio of LC3II/LC3I of BXPC-3-LG cells, accompanying with downregulated AMPKα1 (Fig. 5a and b). Moreover, immunofluorescence staining demonstrated that upregulation of PKM2 led to a reduced number of autophagosome in the cytoplasm of BXPC-3 with hypoglucose treatment (Fig. 5c). On the contrary, knockdown of PKM2 by transfection with siPKM2 further increased the expression of AMPKα1, Beclin1, and density ratio of LC3II/LC3I (Fig. 5d and e), as well as number of autophagosome in the cytoplasm of BXPC-3 with hypoglucose treatment (Fig. 5f). The similar results were observed in PANC-1 cells (SFig. 8).

### Knockdown of AMPKα1 reversed the effects of decreased PKM2 on survival and autophagy of PC cells in hypoglucose condition

**Fig. 6:**
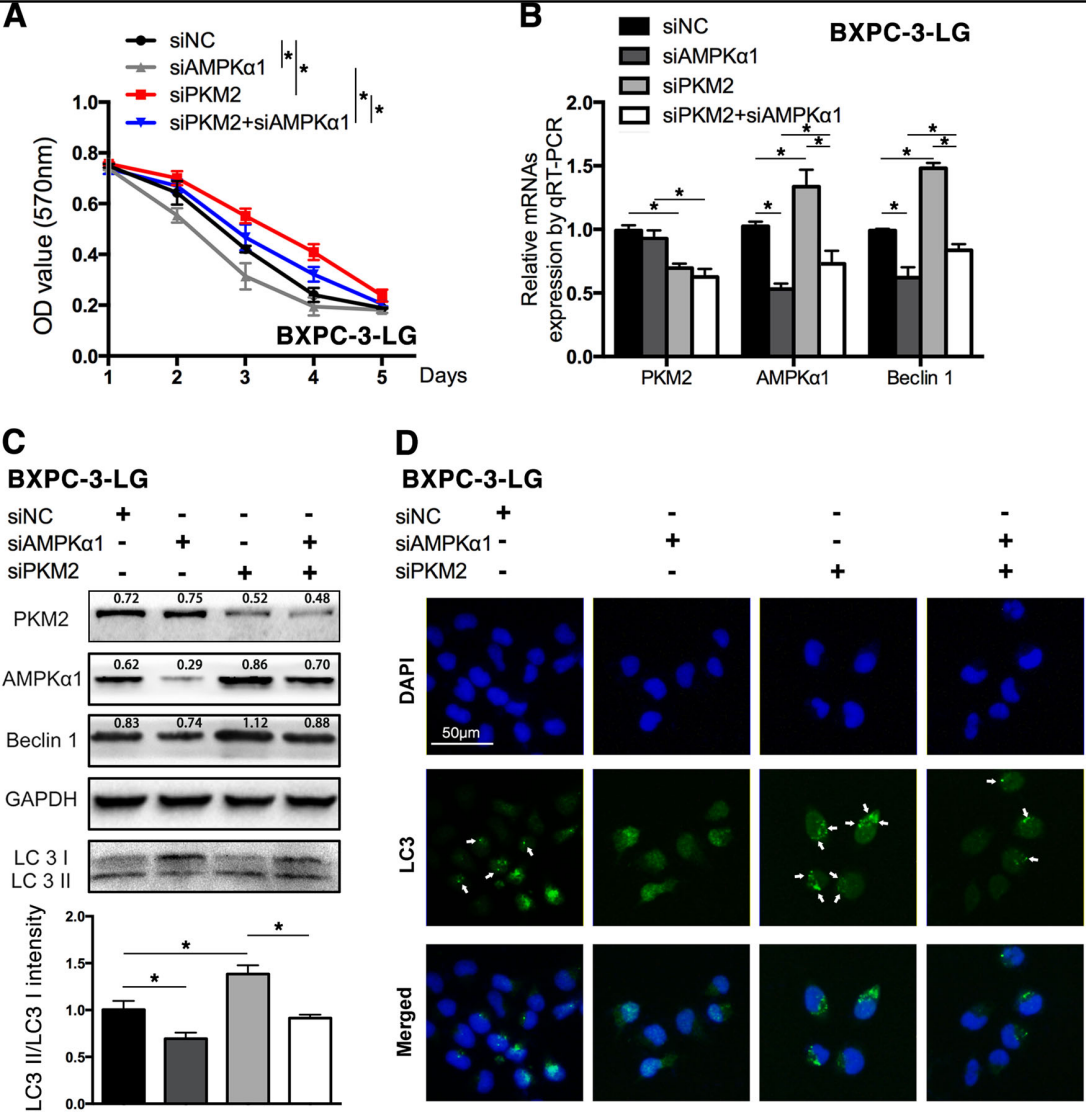
Knockdown of AMPKα1 expression reversed the effects of reduced PKM2 on BXPC-3-LG cells **a** The survival of BXPC-3-LG was tested after transfected with siPKM2, siAMPKα1, and co-transfected with both of them for 5 days. **b** The expression of PKM2, AMPKα1, and Beclin1 was tested by qRT-PCR. **c** PKM2, AMPKα1, and autophagy-related proteins were represented by western blot (upper); the histogram showed the intensity ratio of LC3II/LC3I (lower). **d** Immunofluorescence was performed to test the autophagosomes in cells with the antibody of LC3. The arrow indicates the autophagosomes (*P < 0.05; **P < 0.01)

On account of AMPKα1 playing pivotal roles in energy restriction and autophagy, the present study further explored whether the pro-autophagy role of decreased PKM2 depended on upregulation of AMPKα1. As shown in Fig. 6a, the survival of BXPC-3-LG cells in hypoglucose was decreased after transfection with siAMPKα1, as well that the siPKM2-enhanced survival was also abolished by the co-transfection of siAMPKα1. Moreover, the expression of Beclin1 (Fig. 6b) and the density ratio of LC3II/LC3I were significantly decreased in BXPC-3-LG cells after the downregulation of AMPKα1 (Fig. 6c). After co-transfection with siAMPKα1, the siPKM2 enhanced expression of Beclin1 and the density ratio of LC3II/LC3I was also restrained (Fig. 6b and c). Furthermore, downregulation of AMPKα1 decreased autophagosomes in the BXPC-3-LG cells and also abrogated the siPKM2-enhanced autophagy (Fig. 6d). The similar results were found in PANC-1 cells (SFig. 9).

### AMPKα1 remarkably increased in PC tissues and presents no correlation with PKM2 expression

The upregulation of AMPKα1 in PC tissues has been already identified in our previous research^26^, which was further validated in 24 coupled pancreatic tissues by qRT-PCR (SFig. 10A). Moreover, we uncovered the underlying relationship between AMPKα1 and PKM2 expression in pancreatic tissues. However, no significant correlation between them was observed in either total 48 samples or 24 cancer tissues (SFig. 10B).

## Discussion

Overexpression of PKM2 is broadly described in a variety of cancer tissues^27,28^, while its expression and function in PC are not clearly elucidated. Our present study demonstrated that PKM2 was overexpressed in PC but rarely in adjacent NP tissues, which was coincident with the result of Feng et al.^22^. On the contrary, Aloysius et al.^23^ reported that there was a complete lack of PKM2 expression in PC tissues. After careful comparison, we concluded that the use of a different antibody for detecting PKM2 might be the major factor leading to the discrepancy. As described in Aloysius’s research, the antibody could only detect the active (tetrameric) form of PKM2 but not the total PKM2 expression. According to the findings of Hitosugi et al., active PKM2 could be inhibited by oncogenic fibroblast growth factor receptor type 1 (FGFR1)^29^, which was overexpressed or mutational activated in various human solid tumors including PC^30^. Therefore, negative staining of activated PKM2 in Aloysius’s study might be attributed to the inhibition of active PKM2 by overexpressed FGFR1 in PC tissues. On the contrary, the antibody in our study can detect total PKM2 including active and inactive form by both immunohistochemistry and western blot analysis. Therefore, the specific overexpression of PKM2 in PC tissues could be detected in our study. Moreover, most of our specimens derived from PC was in an advanced stage, while the clinical stage of PC was not clearly clarified in results of Aloysius. The different clinical stage of PC specimens and the racial difference might be the other factors involving the different outcome between others and us. Nevertheless, qRT-PCR results validated that the mRNA level of PKM2 increased in PC tissues.

Interestingly, our results showed that the knockdown of PKM2 did not lead to impairment of PC cell proliferation, which is coincident with the findings of Cortéscros et al.^19^ and Qin et al.^31^. Nevertheless, all of these results imply that PKM2 is heterogeneous and its role is complicated in different cancers. Since PC is a hypovascular tumor with comparably insufficient energy supply, we further investigated the role of PKM2 on PC cells in hypoglucose environment. Significantly, our results showed that both the protein expression and activity of PKM2 were decreased in hypoglucose condition. More than that, we observed that PKM2 knockdown could further contribute to survival of PANC-1 and BxPC-3 cells in hypoglucose condition, but without effects on chemosensitivity and invasion ablity. Thus, these results indicated that the responsively suppressed PKM2 could promote the survival of PC cells in hypoglucose environment.

Because downregulation of PKM2 facilitated survival of PC cells upon hypoglucose, we wonder the possible pathway which was involved in this process. Since AMPKα1, SIRT1, AKT pathways are critical pathways that are involved in the glucose metabolism and proliferation of cancer cells, we first evaluated the expression level of them in further experiment. In the present study, we revealed that p-AMPKα1, SIRT1, and p-AKT were significantly increased in the hypoglucose-treated PC cells. Moreover, the activation of p-AMPKα1, SIRT1, and p-AKT was further increased after knockdown of PKM2. Therefore, our results indicated that the PKM2 knockdown activated AMPKα1, SIRT1, AKT pathway to facilitate survival of PC cells under energy-insufficient condition. Similarly, results from Qin et al. showed that knockdown of PKM2 also led to activation of Akt, which further protects the survival of cancer cells^31^. Interestingly, the PKM2 knockdown in H1299 cells activated AMPK signaling and stimulated mitochondrial biogenesis and autophagy to maintain energy homeostasis^32^.

Moreover, our results showed that downregulation of PKM2 increased NADPH/NADP, but decreased the production of lactic acid, which implies a shifting of glucose flux to PPP for PC cell survival. Activation of PPP results in the accumulation of reductive biosynthesis production in the form of NADPH^33^. NADPH acts as an antioxidant to protect cells from oxidative stress. It reduces glutathione via glutathione reductase, which converts reactive H_2_O_2_ into H_2_O by glutathione peroxidase^34^. Moreover, Anastasiou et al.^33^ demonstrated that the increased reactive oxygen species (ROS) in human lung cancer cells inhibited PKM2, which diverts glucose flux into the PPP for ROS detoxification. Research also showed that hypoglycemia resulted in higher ROS production owing to an imbalance between substrates and oxygen tension^35^. Similarly, our research showed that the ROS level was significantly increased in hypoglucose, which was effectively relieved after PKM2 knockdown. Taken together, these results intensively indicate that the downregulated PKM2 promotes PPP for antioxidants generation to facilitate PC cell survival in hypoglucose.

Recent research has indicated a strong connection between the energy shortage and autophagy. It is a regulated catabolic pathway to digest cellular organelles and macromolecules in order to generate energy^36,37^. Autophagy remains in a very high level in PC cells and plays an important role in carcinogenesis^38,39^. The present study demonstrated that hypoglucose induced remarkable autophagy in PC cells, which was further enhanced by PKM2 downregulation and repressed by PKM2 overexpression. We further explored the mechanism for the regulation of PKM2 on autophagy in hypoglucose condition. There is evidence to support a role for AMPK in autophagy induction in response to glucose starvation. Under glucose starvation, AMPK promotes autophagy by directly activating Ulk1 through phosphorylation of Ser 317 and Ser 777^40^. Results from Mihaylova and Shaw^41^ also showed that LKB–AMPK pathway-dependent phosphorylation of p27 at Thr 198 stabilizes p27 and permits cells to survive growth factor withdrawal and metabolic stress through autophagy^41^. SIRT1 and AMPKα1 are well recognized as autophagy promoters, especially in glucose-deprivation condition according to the study of Chang^42^. On the other hand, recent evidence indicates that the PI3K–Akt–mTOR pathway leads to suppression of autophagy^43^. Nevertheless, research from Aleksandar et al.^44^ demonstrated that AMPK controls osteogenic differentiation of human mesenchymal stem cells through both early mTOR inhibition-mediated autophagy and late activation of Akt/mTOR signaling axis, which indicates a crosstalk between AMPK and Akt pathway^44^. Therefore, since AMPK and AKT possess opposite effect on autophagy, the activation of both AMPK and AKT pathway in glucose starvation indicated that the hypoglucose-induced autophagy is a complex compromise of various signaling pathways. Furthermore, knockdown of AMPKα1 remarkably prohibited survival of PC cells and decreased the autophagy which was induced by hypoglucose and PKM2 knockdown. Therefore, this result of rescue experiment about autophagy implied that PKM2–AMPK pathway might play more important role in autophagy regulation under hypoglucose condition.

Taken together, our present work implicates that PKM2 is responsively downregulated in hypoglucose environment to increase metabolic flux via the PPP and AMPKα1-dependent autophagy, which contributes to the survival of PC cells. Nevertheless, provided numerous inconsistent results have been demonstrated from different studies, the multifaceted functions of PKM2 in tumorigenesis of various cancers need further extensive research.

## Materials and methods

### Patients and tissue samples

PC tissues and matched adjacent NP tissues were collected from 24 primary PC patients at Pancreatic Surgery Center, Union Hospital (Wuhan, China). The complete details of the entire study design and procedures involved were in accordance with the Declaration of Helsinki. All participants and their parents gave their written informed consent to participate in the study after the risks and benefits we discussed in detail. The ethics committee of the Union Hospital approved this study.

### Cell culture

PC cell lines, PANC-1 and BXPC-3, were bought from American Type Culture Collection. They were tested and authenticated for genotypes by DNA fingerprinting. These cell lines were passaged for less than 6 months after resuscitation, and no reauthorization was done. Cells were incubated in 5% CO_2_ at 37℃ with the complete medium, which was composed of 90% RPMI-1640, 10% fetal bovine serum, and 100 U/ml penicillin and streptomycin. Twenty-four hours post-transfection with siPKM2, we replaced with 0.5 mM glucose containing RPMI medium as a hypoglucose treatment^45^.

### Western blot analysis and immunohistochemistry

Western blot analysis and immunohistochemistry have been carried out as described previously^33^. Antibodies for research were below: rabbit anti-PKM2, rabbit anti-Beclin1, rabbit anti-LC3, rabbit anti-p-AKT, and mouse/rabbit secondary antibody were purchased from Cell Signaling Technology; rabbit anti-AMPKα1, rabbit anti-p-AMPKα1, rabbit anti-SIRT1, and mouse anti-GAPDH were purchased from Santa Cruz Biotechnology. Immunoblotting of LC3 usually gives two bands: LC3-I and LC3-II. The LC3-II/LC3-I ratio correlates with the number of autophagosomes and is applied as the marker for autophagic maker^34^. For immunohistochemistry analysis, we considered PKM2-positive expression when cases with more than 30% cancer cells were stained.

### Immunofluorescence

LC3 (Cell Signaling Technology), CD31 (Goodbio Technology Co., Ltd, China), and PKM2 (Cell Signaling Technology) for immunofluorescence were diluted into 1:200, 1:50, and 1:200 respectively. For microvessel density measurement, the intensity of CD31 fluorescence was determined by measuring IOD. Image pro plus was used to identify the intensity and area of fluorescence. All pictures were divided into two groups according to the mean intensity value of CD31 (hypervascular and hypovascular).

### RNA interference and plasmid transfection

SiPKM2 was bought from Santa Cruz Biotechnology, siAMPKα1 and corresponding negative control (NC) were purchased from Guangzhou Ribobio Co., PKM2 overexpressed plasmid and corresponding negative control were purchased from Shanghai GeneChem Co., SiRNAs were transfected with Lipofectamine 2000 at a final concentration of 50 nM, while plasmids were transfected with 0.2 μg for 96-well plate and 1.6 μg for 12-well plate. The effects of the transient transfection were supported by qRT-PCR and western blot analysis.

### Quantitative real-time reverse transcription polymerase chain reaction

Total RNA was extracted from tissues with Trizol reagent according to the suppliers’ protocol. The mRNAs were reverse transcribed by using the PrimeScript^®^ RT Master Mix Perfect Real Time and followed by qRT-PCR analysis with SYBR Premix Ex Taq II. The expression levels of PKM2, AMPKα1, and Beclin1 were normalized to GAPDH with the primers listed in Supplementary Table S1 and all reactions were performed in triplicate.

### Cell proliferation assay and drug resistance

The proliferation capacity was measured by using the methyl-thiazolyltetrazolium (MTT) assay. Transfected cells were placed in a 96-well plate and observed for 5 days. Cells were cultured with 20 μl MTT (5 mg/ml) per well, then replaced with 150 μl DMSO, and further determined the absorbance by an ELISA reader at 570 nm. Cell sensitivity to 5-Fluorouracil and Gemcitabine was also maintained with the MTT assay. Transfected cells were treated with various concentrations of 5-Fluorouracil or Gemcitabine on the second day. All MTT assay were repeated three times in six replicates.

### Cell viability

2.5 × 10^3^ cells per well were placed for 96-well plates in six replicates. Twenty-four hours later, cells were transfected with PKM2 siRNA and negative control at a final concentration of 50 nM, respectively. Afterwards, cells were treated with various glucose concentrations containing the medium for another 2 days and then cell viability values determined by MTT assay. The experiment was performed with three replications.

### Invasion assay

We utilized the Corning Matrigel Invasion Chamber of pore size 8 μm to detect the cell invasion capacity. Chambers were coated by 1:9 diluted Matrigel, and the medium containing 0.1% serum was placed in the upper chamber while lower chambers were filled with medium containing 30% serum. Fixed and stained after 48 h. The cells on the lower surface of the membrane were then counted under a microscope in nine fields.

### Pyruvate kinase activity, lactic acid, and NADPH/NADP assays

Pyruvate kinase assay kit (A076-1; Nanjing Jiancheng Bioengineering Institute, Jiangsu, China), Lactic Acid assay kit (A109-2; Nanjing Jiancheng Bioengineering Institute, Jiangsu, China), and NADPH/NADP Quantification Colorimetric Assay Kit (BioVision, Milpitas, CA, USA) were all purchased from the company. Two days after transfection, cells were collected and treated according to the manual protocol. The pyruvate kinase activity, lactic acid concentration, and NADPH/NADP ratio were detected through the absorbance at 340, 530, and 450 nm by an ELISA reader, respectively.

### Intercellular ROS assay

The level of intracellular ROS was determined using dichlorodihydrofluorescein diacetate assay (DCFH-DA; Beyotime Biotechnology, China). Briefly, treated pancreatic cells were washed with phosphate-buffered saline (PBS) and incubated with 10 μM of DCFH-DA for half an hour. Subsequently, the cells were washed with PBS again. DCF fluorescence was observed by using a scanning fluorescence microscope.

### Statistical analysis

Data were analyzed using the SPSS 13.0 software (*SPSS*, Chicago, IL) and all values were expressed as Mean ± SD. Comparison between two groups was performed using unpaired Student’s *t*-test while ANOVA was used to identify difference among groups. Statistical significance was noted at *p* < 0.05. Three independent triplicated experiments were performed for cell biological assays.

## Electronic supplementary material

Supplementary figure legends

Supplementary Figure 1. Knockdown of PKM2 had no significant effects on proliferation, chemoresistance or invasion of pancreatic cancer cells

Supplementary Figure 2. Downregulation of PKM2 failed to regulate the capacity of chemoresistance and cell invasion in pancreatic cancer cells under hypoglucose condition

Supplementary Figure 3. Overexpression of PKM2 downregulated metabolism associated protein, promoted lactic acid generation, suppressed PPP and increased ROS accumulation in hypoglucose treatment in B

Supplementary Figure 4. Downregulation of PKM2 upregulated metabolism associated protein, reduced lactic acid generation, activated PPP and suppressed ROS accumulation in hypoglucose treatment in BXPC

Supplementary Figure 5. Overexpression of PKM2 downregulated metabolism associated protein, promoted lactic acid generation, suppressed PPP and increased ROS accumulation in hypoglucose treatment in P

Supplementary Figure 6. Downregulation of PKM2 upregulated metabolism associated protein, reduced lactic acid generation, activated PPP and suppressed ROS accumulation in hypoglucose treatment in PANC

Supplementary Figure 7. Hypoglucose treatment induced autophagy in PANC-1

Supplementary Figure 8. Decreased PKM2 promoted autophagy of PANC-1 cells in hypoglucose by upregulating AMPKα1 expression

Supplementary Figure 9. Knockdown of AMPKα1 expression reversed the effects of decreased PKM2 on PANC-1-LG cells

Supplementary Figure 10. AMPKα1 was overexpressed in human pancreatic cancer tissues but showed no correlation with PKM2
