# Supplementary material for: The responsively decreased PKM2 facilitates the survival of pancreatic cancer cells in hypoglucose
Source: Cell Death Dis. 2018 Jan 26;9(2):133. doi: 10.1038/s41419-017-0158-5 (PMC5833844; doi:10.1038/s41419-017-0158-5)
Supplement: Supplementary file 1 — Supplementary figure legends [file 41419_2017_158_MOESM1_ESM.docx]

**Supplementary Figure 1. Knockdown of PKM2 had no significant effects on proliferation, chemoresistance or invasion of pancreatic cancer cells**

(A) The expression of PKM2 was tested by qRT-PCR and western blot. (B) Activity of PKM2 was tested after transfection with siPKM2 or corresponding negative control (siNC). (C) MTT assays were performed to determine the effect of siPKM2 transfection on the proliferation of pancreatic cancer cells. (D) The cell invasion ability was measured by using Transwell migration chambers. (E) The effects of PKM2 downregulation on chemoresistance of cells to 5-Fluorouracil (5-FU) and Gemcitabine was analyzed with MTT. (*P<0.05; **P<0.01).

**Supplementary Figure 2. Downregulation of PKM2 failed to regulate the capacity of chemoresistance and cell invasion in pancreatic cancer cells under hypoglucose condition**

1. The cell invasion ability under hypoglucose treatment with PKM2 downregulation was measured by using Transwell migration chambers. (B) The effects of PKM2 downregulation on chemoresistance of cells to 5-Fluorouracil (5-FU) and Gemcitabine under hypoglucose treatment was analyzed with MTT. (*P<0.05; **P<0.01)

**Supplementary Figure 3. Overexpression of PKM2 downregulated metabolism associated protein, promoted lactic acid generation, suppressed PPP and increased ROS accumulation in hypoglucose treatment in BXPC-3**

(A) Western blot was performed to identify the expression of p-AMPKα1, AMPKα1, p-AKT and SIRT1 after upregulation of PKM2. Relative intensity value is marked. (B) The concentration of lactic acid was tested 48 hours post transfection with pCDNA-PKM2 in hypoglucose condition. (C) The ratio of NADPH/NADP was measured after PKM2 overexpression under hypoglucose treatment. (D) Cells were labeled by DCFH-DA (10μM) and examined using fluorescence microscope to reveal the level of oxidative stress after transfection with pCDNA-PKM2. Average intensity was listed at the upright angle of the image. (*P<0.05; **P<0.01)

**Supplementary Figure 4. Downregulation of PKM2 upregulated metabolism associated protein, reduced lactic acid generation, activated PPP and suppressed ROS accumulation in hypoglucose treatment in BXPC-3**

(A) Western blot was performed to identify the expression of p-AMPKα1, AMPKα1, p-AKT and SIRT1 after siPKM2 transfection. Relative intensity value is marked. (B) The concentration of lactic acid was tested 48 hours post transfection with siPKM2 in hypoglucose condition. (C) The ratio of NADPH/NADP was measured after PKM2 downregulation under hypoglucose treatment. (D) Cells were labeled by DCFH-DA (10μM) and examined using fluorescence microscope to reveal the level of oxidative stress after transfection with siPKM2. Average intensity was listed at the upright angle of the image. (*P<0.05; **P<0.01)

**Supplementary Figure 5. Overexpression of PKM2 downregulated metabolism associated protein, promoted lactic acid generation, suppressed PPP and increased ROS accumulation in hypoglucose treatment in PANC-1**

1. Western blot was performed to identify the expression of p-AMPKα1, AMPKα1, p-AKT and SIRT1 after upregulation of PKM2. Relative intensity value is marked. (B) The concentration of lactic acid was tested 48 hours post transfection with pCDNA-PKM2 in hypoglucose condition. (C) The ratio of NADPH/NADP was measured after PKM2 overexpression under hypoglucose treatment. (D) Cells were labeled by DCFH-DA (10μM) and examined using fluorescence microscope to reveal the level of oxidative stress after transfection with pCDNA-PKM2. Average intensity was listed at the upright angle of the image. (*P<0.05; **P<0.01)

**Supplementary Figure 6. Downregulation of PKM2 upregulated metabolism associated protein, reduced lactic acid generation, activated PPP and suppressed ROS accumulation in hypoglucose treatment in PANC-1**

(A) Western blot was performed to identify the expression of p-AMPKα1, AMPKα1, p-AKT and SIRT1 after siPKM2 transfection. Relative intensity value is marked. (B) The concentration of lactic acid was tested 48 hours post transfection with siPKM2 in hypoglucose condition. (C) The ratio of NADPH/NADP was measured after PKM2 downregulation under hypoglucose treatment. (D) Cells were labeled by DCFH-DA (10μM) and examined using fluorescence microscope to reveal the level of oxidative stress after transfection with siPKM2. Average intensity was listed at the upright angle of the image. (*P<0.05; **P<0.01)

**Supplementary Figure 7. Hypoglucose treatment induced autophagy in PANC-1**

(A) QRT-PCR tested the mRNA of Beclin1 in PANC-1 treated with hypoglucose medium for 0h, 24h, 48h and 72h. (B) Moreover, the result of western blot showed the expression of AMPKα1, Beclin1 and LC3II/LC3I. Relative intensity value is marked. (C) Treatment of PANC-1 with hypoglucose showed increased autophagosomes in cytoplasma as indicated by the arrow. (*P<0.05; **P<0.01)

**Supplementary Figure 8. Decreased PKM2 promoted autophagy of PANC-1 cells in hypoglucose by upregulating AMPKα1 expression**

(A) The expression level of PKM2, AMPKα1 and Beclin1 was tested by qRT-PCR 48h after transfection with pCDNA-PKM2 in PANC-1-LG. (B) The expression of PKM2, AMPKα1 and autophagy related protein was determined by western blot 48h after transfection with pCDNA-PKM2 in PANC-1-LG. Relative intensity value is marked. (C) The results of immunofluorescence showed that the autophagy of pCDNA-PKM2 transfected group was enhanced in hypoglucose condition. The autophagosome was indicated with the arrow. (D) The expression level of PKM2, AMPKα1 and Beclin1 was tested by qRT-PCR 48h after transfection with siPKM2 in PANC-1-LG. (E) The expression of PKM2, AMPKα1 and autophagy related protein was determined by western blot 48h after transfection with siPKM2 in PANC-1-LG. Relative intensity value is marked. (F) The results of immunofluorescence showed that the autophagy of siPKM2 transfected group was enhanced in hypoglucose condition. The autophagosome was indicated with the arrow. (*P<0.05; **P<0.01)

**Supplementary Figure 9. Knockdown of AMPKα1 expression reversed the effects of decreased PKM2 on PANC-1-LG cells**

1. The survival of PANC-1-LG was tested after transfected with siPKM2, siAMPKα1 and co-transfected with both of them for 5 days. (B) The expression of PKM2, AMPKα1 and Beclin1 was tested by qRT-PCR 48h after transfection. (C) PKM2, AMPKα1 and autophagy related proteins were represented by western blot (upper), the histogram showed the intensity ratio of LC3II/LC3I (lower). Relative intensity value is marked. (D) Immunofluorescence was performed to test the autophagosomes in cells with the antibody of LC3. The arrow indicates the autophagosomes. (*P<0.05; **P<0.01)

**Supplementary Figure 10. AMPKα1 was overexpressed in human pancreatic cancer tissues but showed no correlation with PKM2**

1. (A) QRT-PCR was used to identify the expression of AMPKα1 mRNA in human pancreatic cancer tissues (PC) and adjacent normal pancreatic tissues (NP). (B) The correlation between AMPKα1 and PKM2 in total 48 human pancreatic tissues and 24 pancreatic cancer tissues were analyzed and no correlation was observed. (*P<0.05; **P<0.01)
