# Supplementary figures and images for: The responsively decreased PKM2 facilitates the survival of pancreatic cancer cells in hypoglucose
Source: Cell Death Dis. 2018 Jan 26;9(2):133. doi: 10.1038/s41419-017-0158-5 (PMC5833844; doi:10.1038/s41419-017-0158-5)

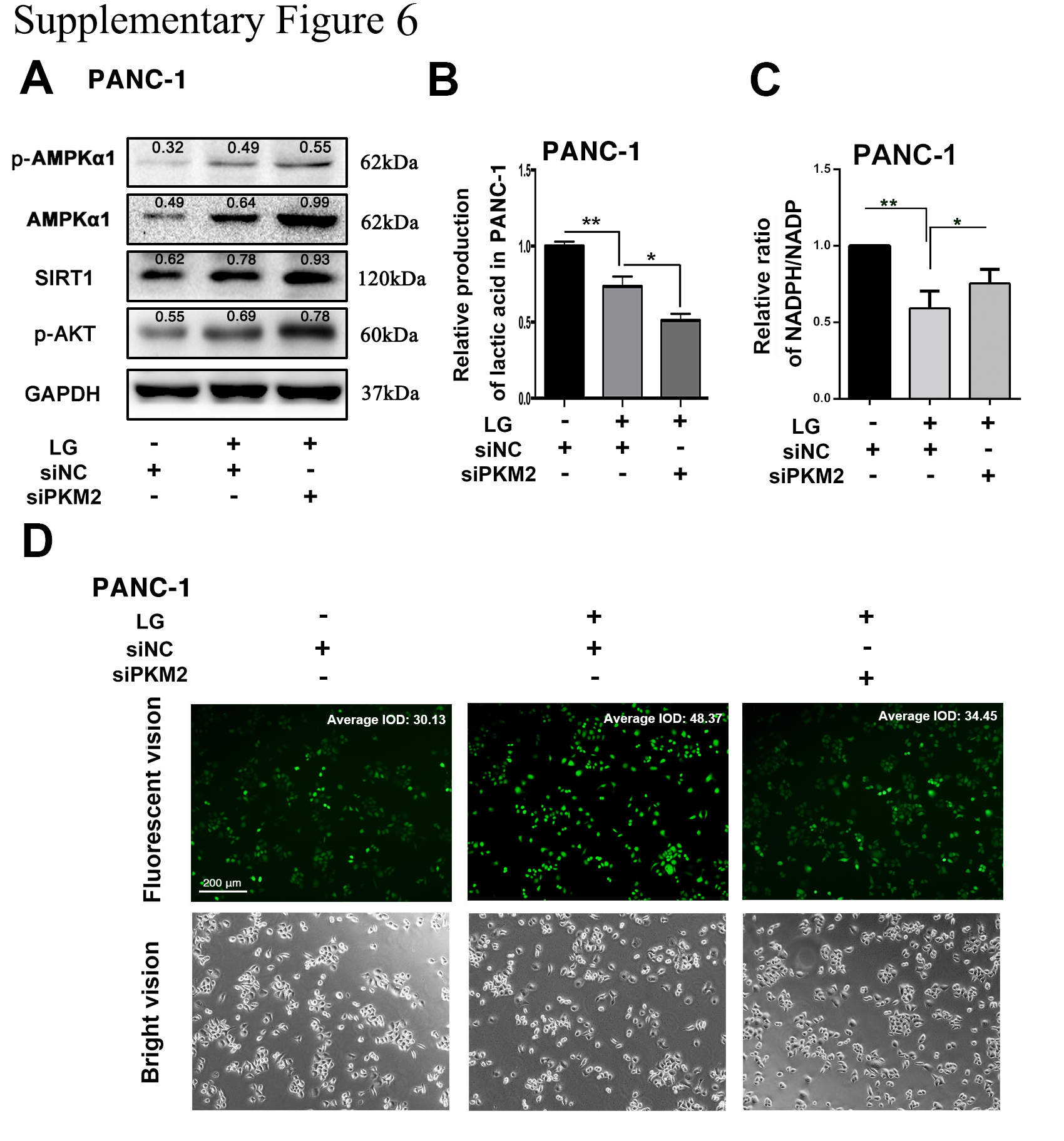

Supplement: Supplementary file 2 — Supplementary Figure 1. Knockdown of PKM2 had no significant effects on proliferation, chemoresistance or invasion of pancreatic cancer cells [file 41419_2017_158_MOESM2_ESM.jpg]

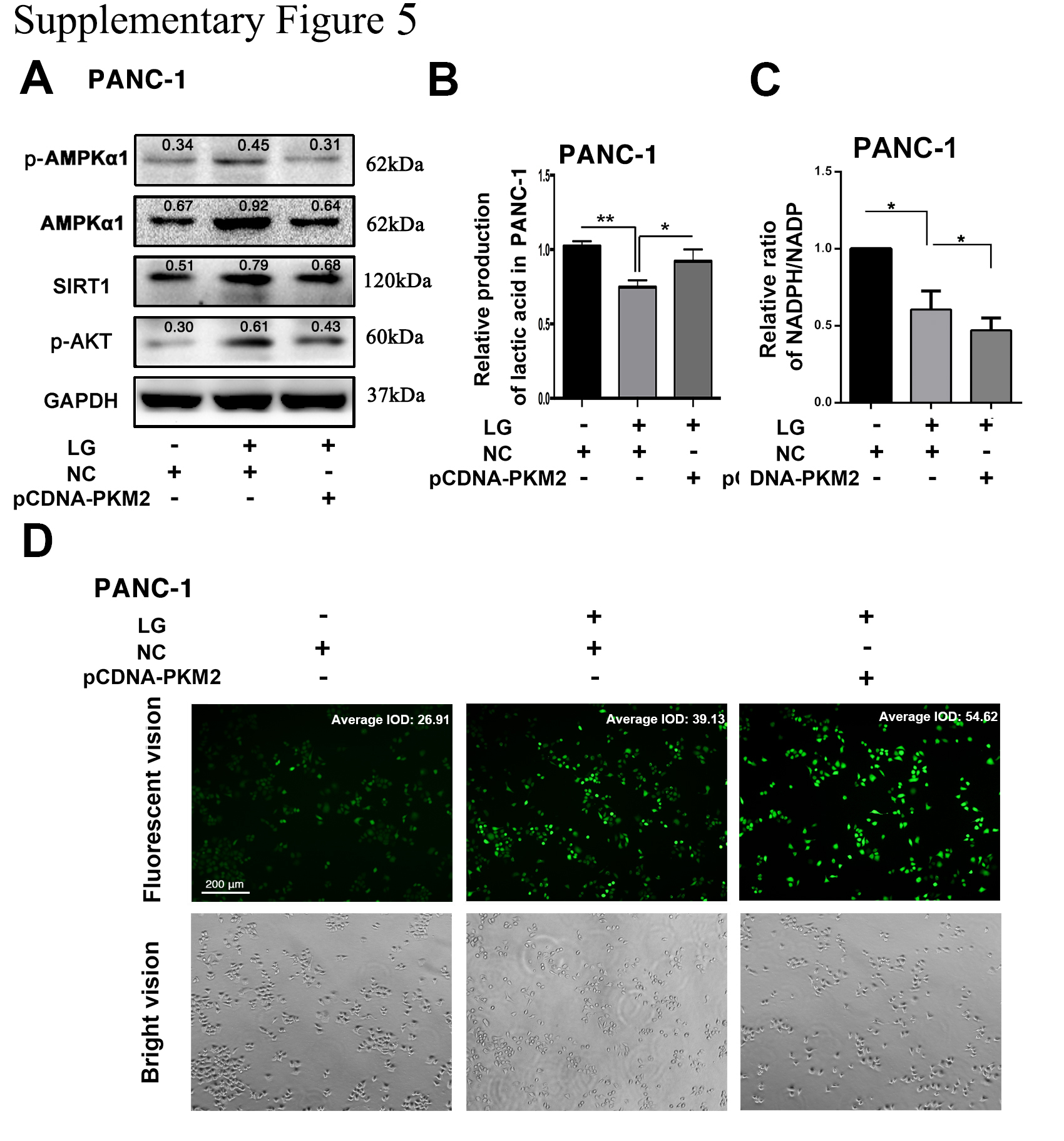

Supplement: Supplementary file 3 — Supplementary Figure 2. Downregulation of PKM2 failed to regulate the capacity of chemoresistance and cell invasion in pancreatic cancer cells under hypoglucose condition [file 41419_2017_158_MOESM3_ESM.jpg]

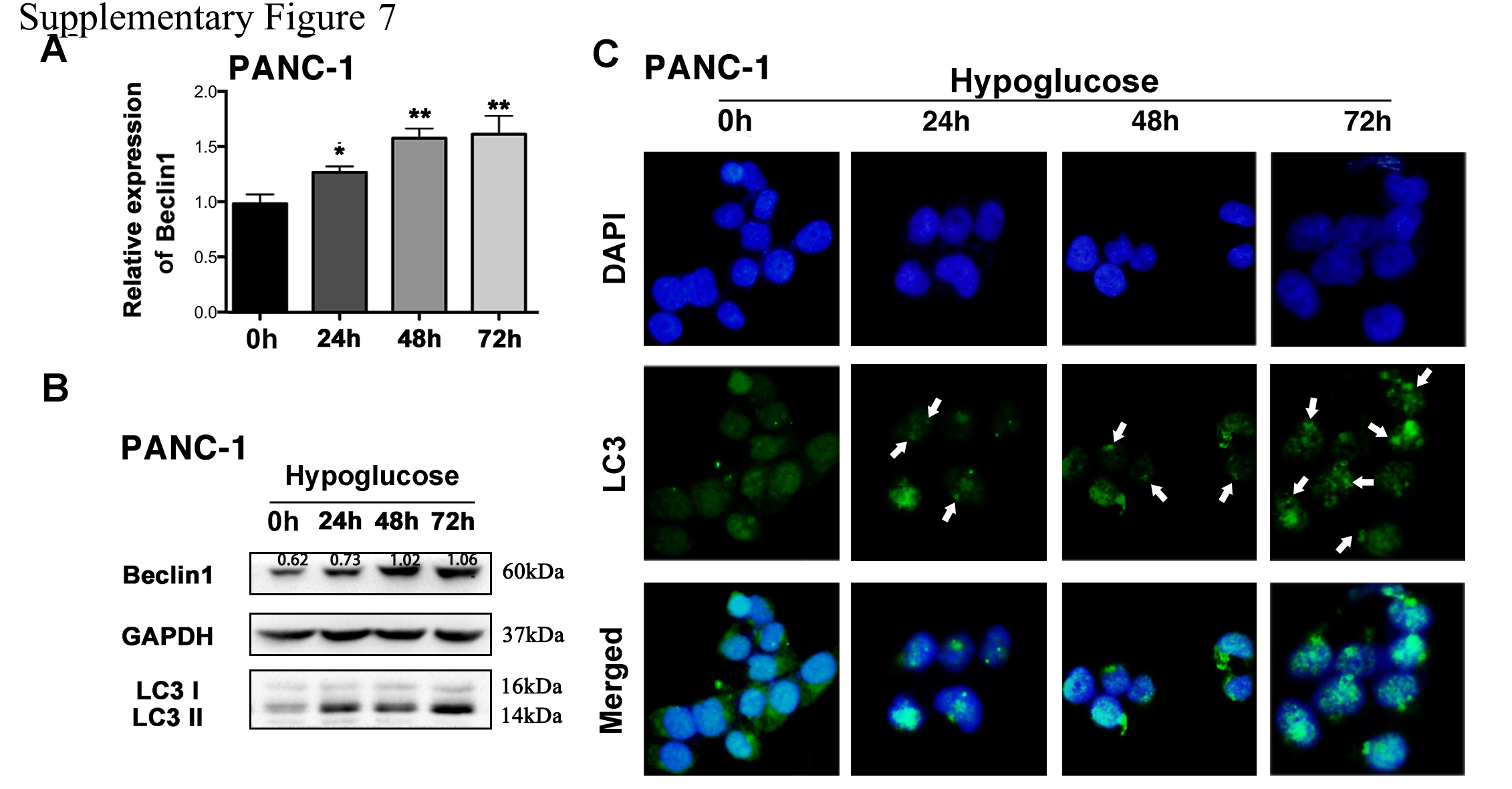

Supplement: Supplementary file 4 — Supplementary Figure 3. Overexpression of PKM2 downregulated metabolism associated protein, promoted lactic acid generation, suppressed PPP and increased ROS accumulation in hypoglucose treatment in B [file 41419_2017_158_MOESM4_ESM.jpg]

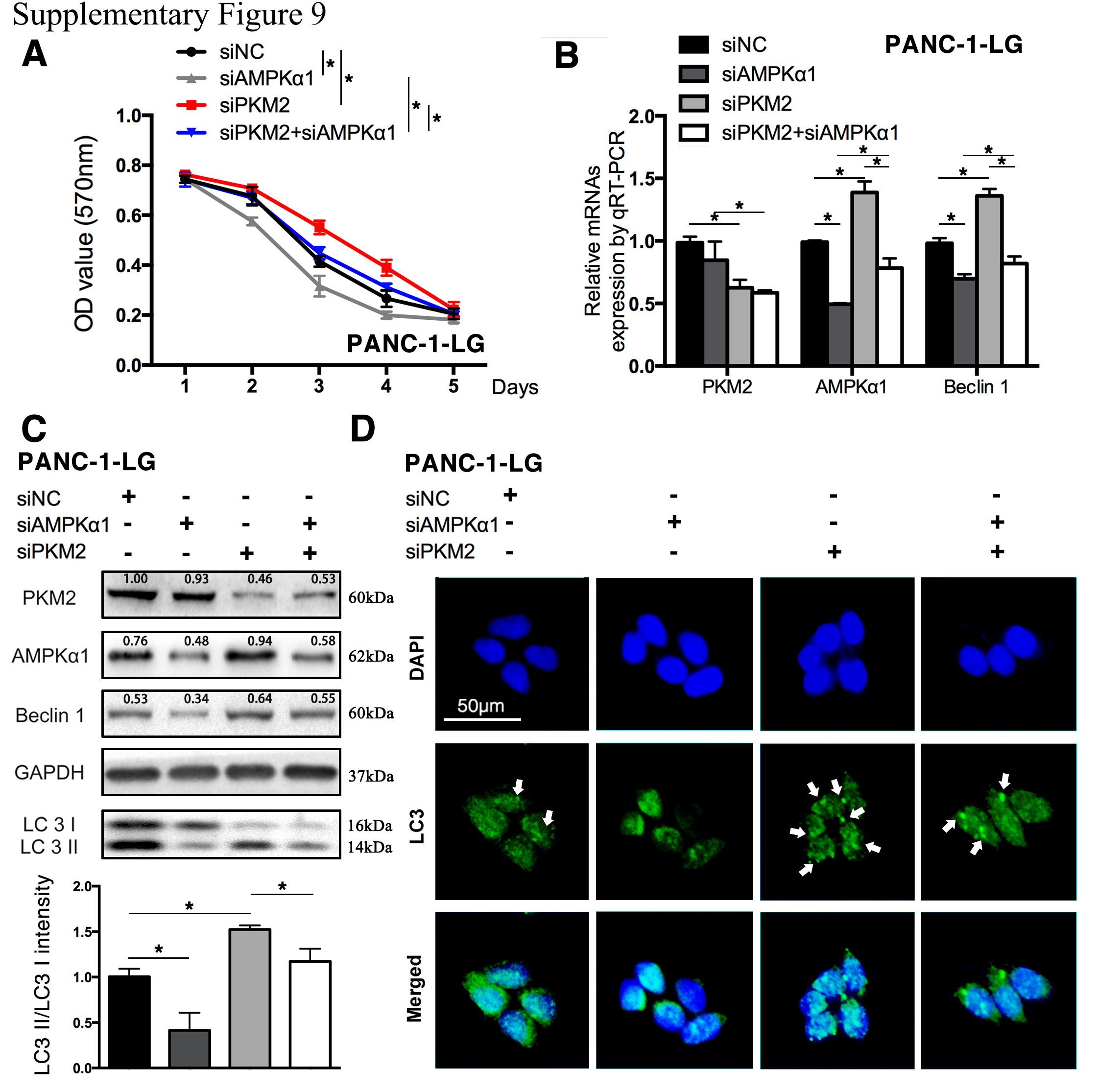

Supplement: Supplementary file 5 — Supplementary Figure 4. Downregulation of PKM2 upregulated metabolism associated protein, reduced lactic acid generation, activated PPP and suppressed ROS accumulation in hypoglucose treatment in BXPC [file 41419_2017_158_MOESM5_ESM.jpg]

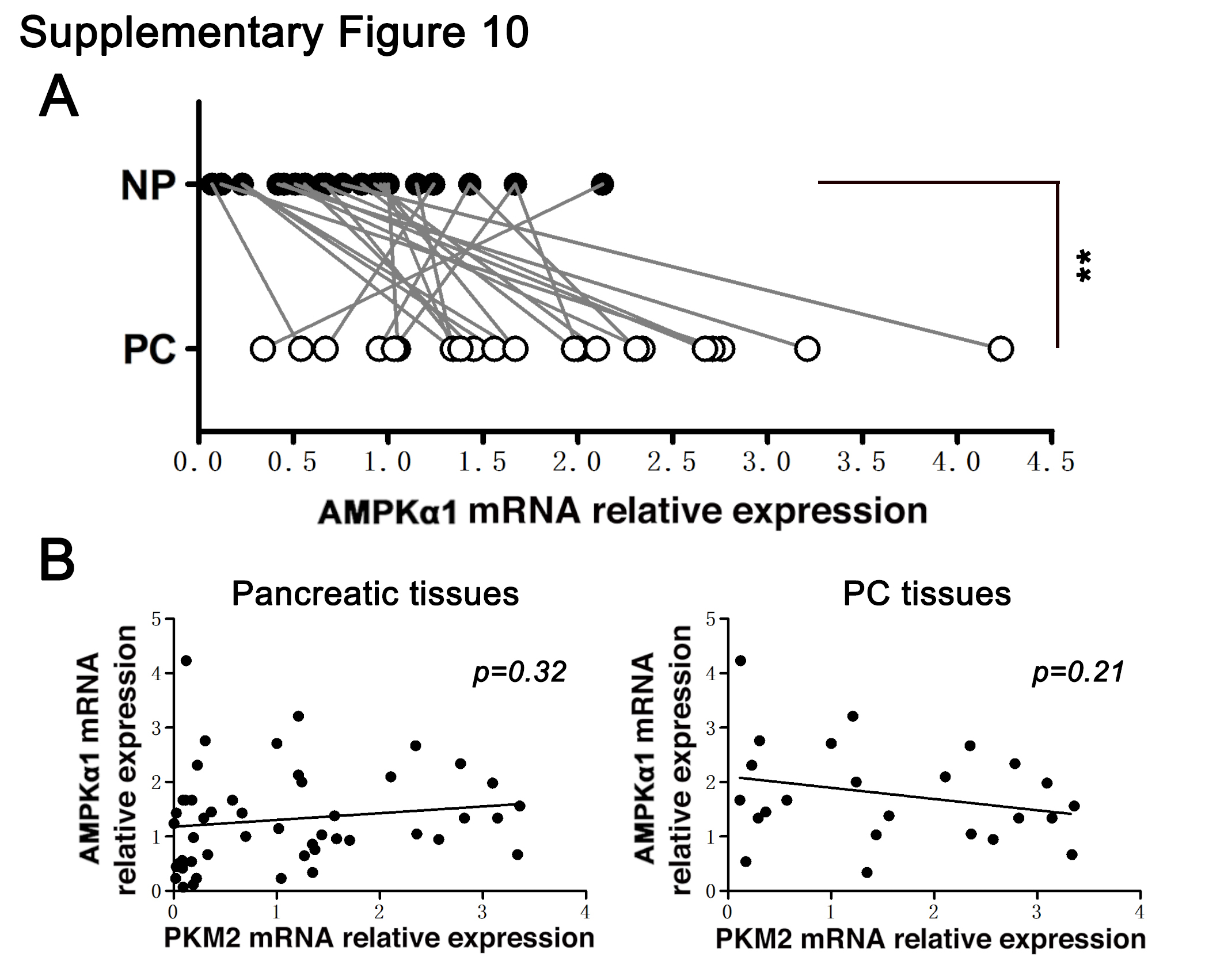

Supplement: Supplementary file 6 — Supplementary Figure 5. Overexpression of PKM2 downregulated metabolism associated protein, promoted lactic acid generation, suppressed PPP and increased ROS accumulation in hypoglucose treatment in P [file 41419_2017_158_MOESM6_ESM.jpg]

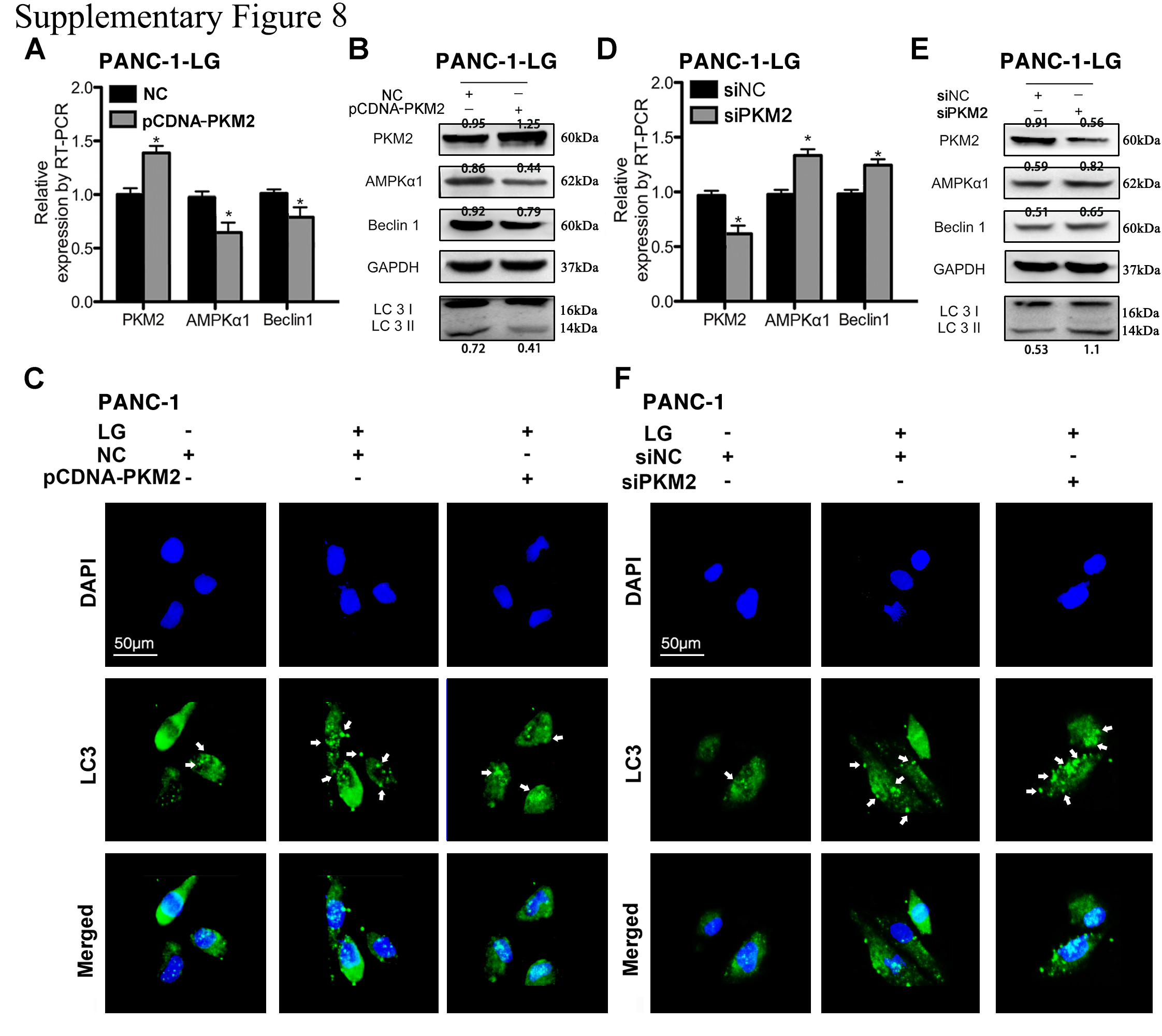

Supplement: Supplementary file 7 — Supplementary Figure 6. Downregulation of PKM2 upregulated metabolism associated protein, reduced lactic acid generation, activated PPP and suppressed ROS accumulation in hypoglucose treatment in PANC [file 41419_2017_158_MOESM7_ESM.jpg]

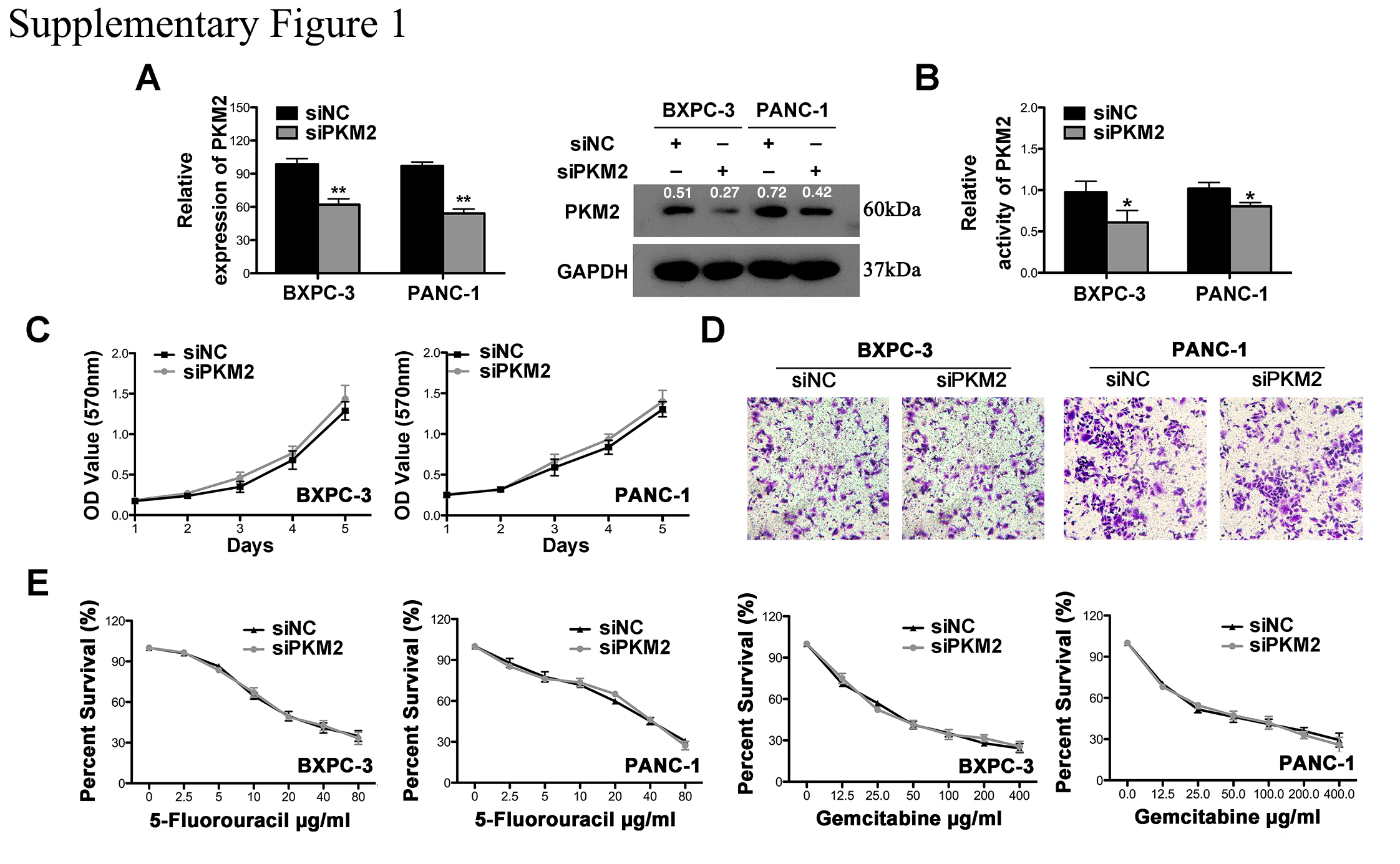

Supplement: Supplementary file 8 — Supplementary Figure 7. Hypoglucose treatment induced autophagy in PANC-1 [file 41419_2017_158_MOESM8_ESM.jpg]

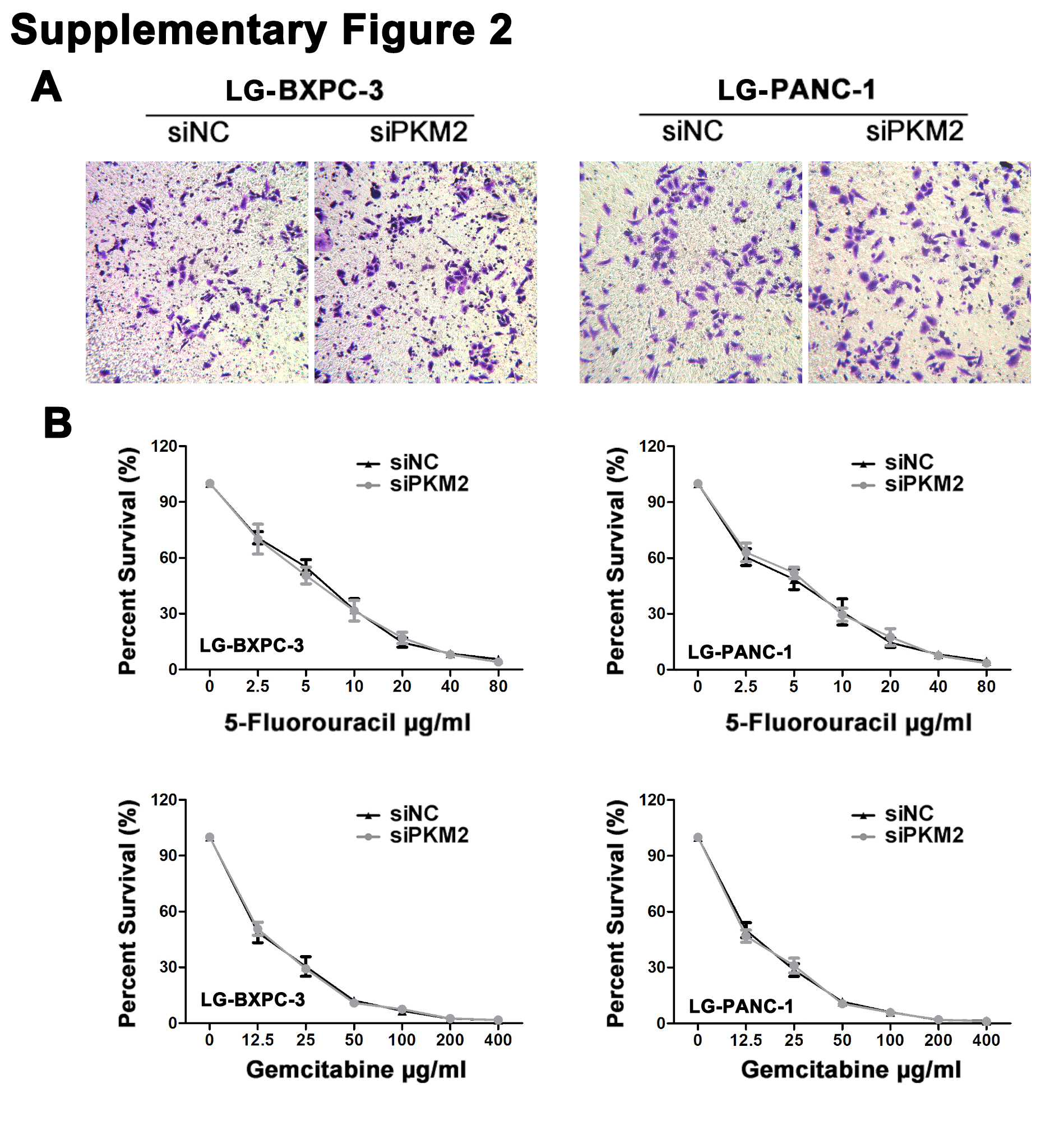

Supplement: Supplementary file 9 — Supplementary Figure 8. Decreased PKM2 promoted autophagy of PANC-1 cells in hypoglucose by upregulating AMPKα1 expression [file 41419_2017_158_MOESM9_ESM.jpg]

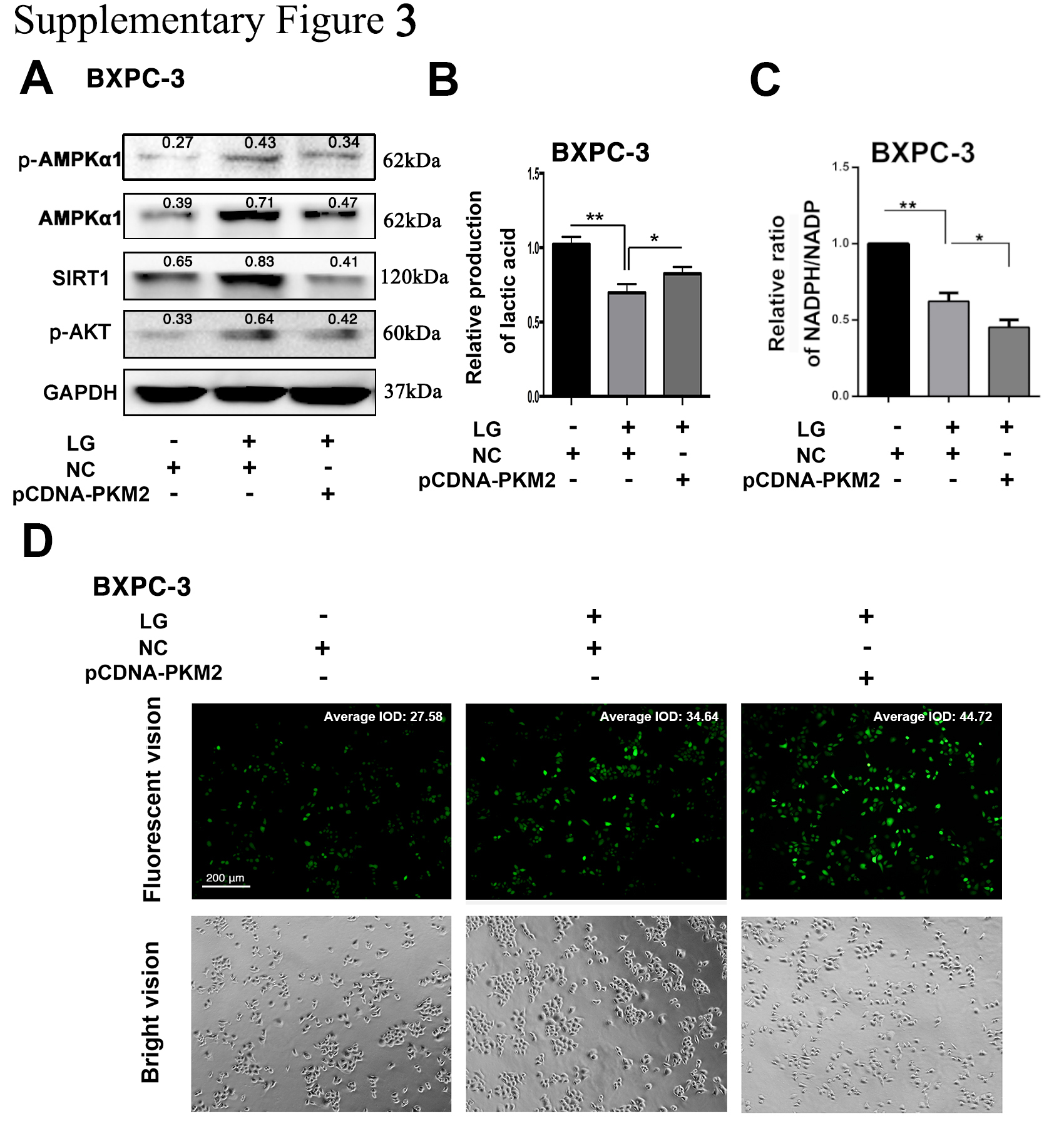

Supplement: Supplementary file 10 — Supplementary Figure 9. Knockdown of AMPKα1 expression reversed the effects of decreased PKM2 on PANC-1-LG cells [file 41419_2017_158_MOESM10_ESM.jpg]

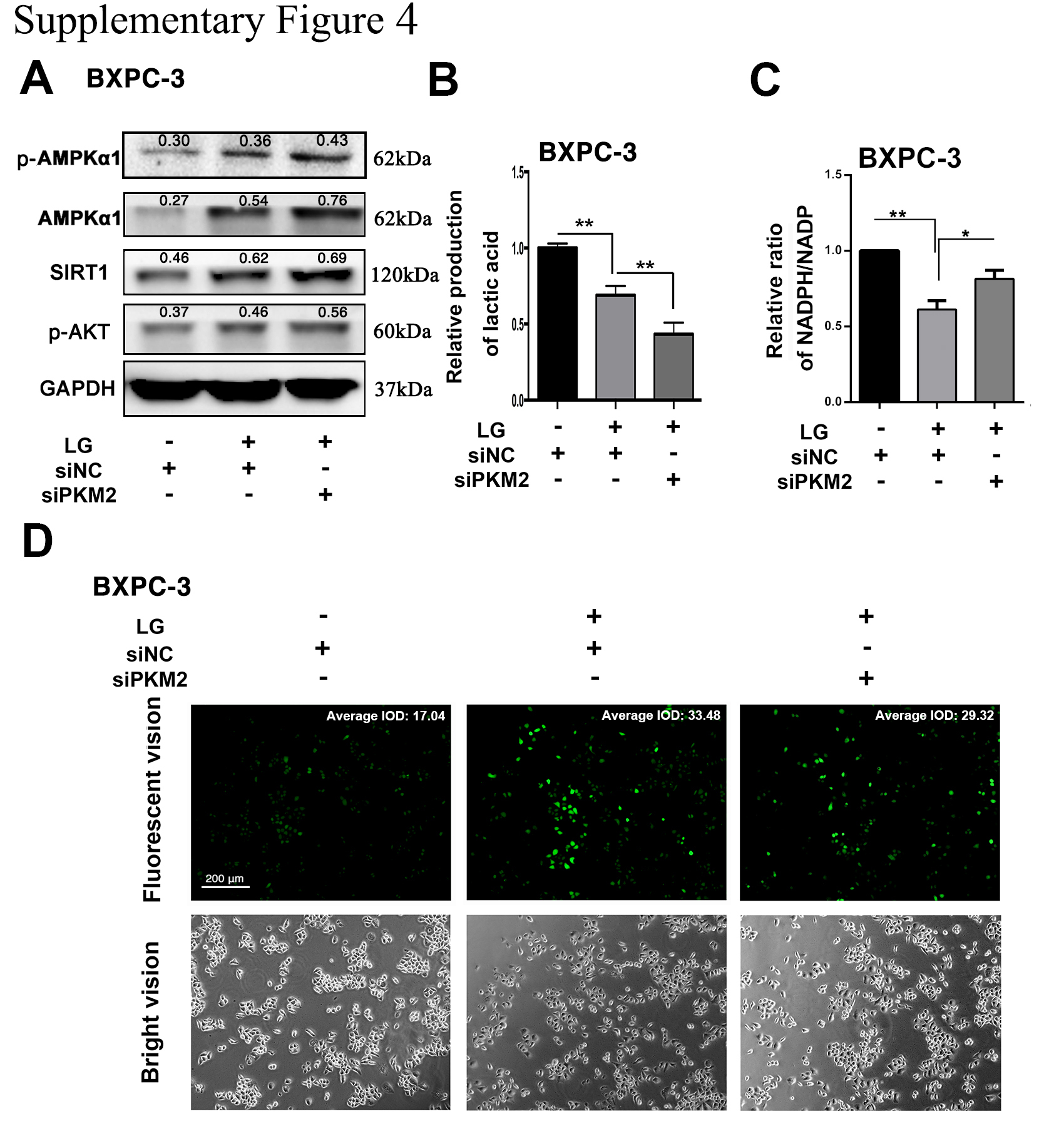

Supplement: Supplementary file 11 — Supplementary Figure 10. AMPKα1 was overexpressed in human pancreatic cancer tissues but showed no correlation with PKM2 [file 41419_2017_158_MOESM11_ESM.jpg]
